# Cell-to-cell spread and massive vacuole formation after *Cryptococcus neoformans *infection of murine macrophages

**DOI:** 10.1186/1471-2172-8-16

**Published:** 2007-08-16

**Authors:** Mauricio Alvarez, Arturo Casadevall

**Affiliations:** 1 Department of Microbiology and Immunology, Albert Einstein College of Medicine, Bronx, NY, 10461, USA

## Abstract

**Background:**

The interaction between macrophages and *Cryptococcus neoformans *(Cn) is critical for containing dissemination of this pathogenic yeast. However, Cn can either lyse macrophages or escape from within them through a process known as phagosomal extrusion. Both events result in live extracellular yeasts capable of reproducing and disseminating in the extracellular milieu. Another method of exiting the intracellular confines of cells is through host cell-to-cell transfer of the pathogen, and this commonly occurs with the human immuno-deficiency virus (HIV) and CD4^+ ^T cells and macrophages. In this report we have used time-lapse imaging to determine if this occurs with Cn.

**Results:**

Live imaging of *Cryptococcus neoformans *interactions with murine macrophages revealed cell-to-cell spread of yeast cells from infected donor cells to uninfected cells. Although this phenomenon was relatively rare its occurrence documents a new capacity for this pathogen to infect adjacent cells without exiting the intracellular space. Cell-to-cell spread appeared to be an actin-dependent process. In addition, we noted that cryptococcal phagosomal extrusion was followed by the formation of massive vacuoles suggesting that intracellular residence is accompanied by long lasting damage to host cells.

**Conclusion:**

*C. neoformans *can escape the intracellular confines of macrophages in an actin dependent manner by cell-to-cell transfer of the yeast leading to infection of adjacent cells. In addition, complete extrusion of internalized Cn cells can lead to the formation of a massive vacuole which may be a sign of damage to the host macrophage. These observations document new outcomes for the interaction of *C. neoformans *with host cells that provide precedents for cell biological effects that may contribute to the pathogenesis of cryptococcal infections.

## Background

*Cryptococcus neoformans *is an encapsulated facultative intracellular pathogen and is the etiologic agent of human cryptococcosis. The interaction of Cn with alveolar macrophages is vital for containing the infection [1,2], however, Cn overcomes this initial host defense barrier using a unique pathogenic strategy involving intracellular replication and cytoplasmic accumulation of polysaccharide-containing vesicles, leading to the formation of spacious phagosomes where multiple cryptococcal cells are present [3,4]. Continued intracellular replication eventually leads to lysis of the host macrophage or to extrusion of the cryptococcal phagosome [5-7]. The Cn intracellular pathogenic strategy in macrophages and amoeba is similar, suggesting that the unique mechanism used by this fungus originated for survival strategy against environmental phagocytic predators [8]. Macrophages are known to fuse with each other [9] and this process of cell-cell fusion has been previously documented and been implicated in host viral spread with viruses such as HIV and vaccinia virus where the host-cell actin plays a crucial role in mediating cell to cell transfer [10-12]. In light of the recent findings reported in our lab and elsewhere, demonstrating extrusion of Cn from macrophages [5,6], in this study we report that under certain conditions, subsequent to phagocytosis, Cn can exit the infected macrophage, and without exposing itself to the extracellular milieu it can immediately enter the cytoplasm of an uninfected macrophage, thus allowing Cn to be passed from one macrophage to another in an apparently actin-mediated process. This mechanism of pathogen spreading has not been previously documented with yeast cells thus suggesting a novel mechanism for dissemination by Cn. In addition, extrusion of a high number of Cn cells was followed by the formation of a massive vacuole, implying that macrophages are potentially damaged by their interaction with Cn. These phenomena provide additional documentation for the complexity of Cn interactions with macrophages.

## Results

**Figure 1:**
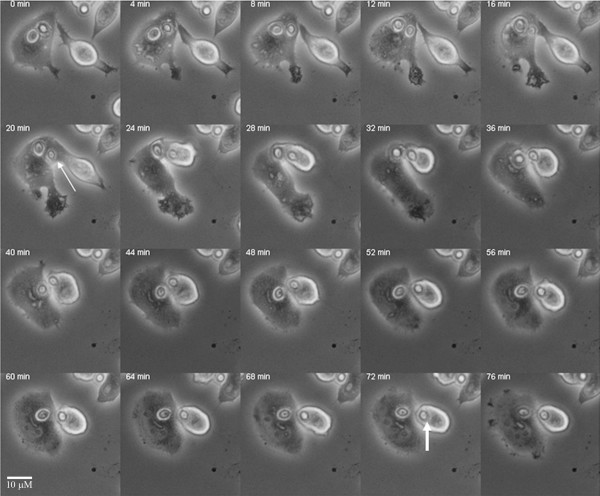
**Cell-to-cell transfer of *C. neoformans *leads to infection of previously uninfected cell**. Following phagocytosis, macrophages closely apposed to each other underwent fusion leading to cell to cell transfer of Cn. The thin arrow indicates the Cn yeast that is being transferred while the wide arrow points to the Cn yeast that has been fully transferred to the previously uninfected murine macrophage cell. Images were collected at 20×.

All Cn strains were efficiently internalized (data not shown) and replicated intracellularly unless heat-killed. Following phagocytosis, and using time-lapse microscopy we witnessed an infrequent (occurring in about 2% of infected macrophages) but previously unknown event where an infected macrophage transferred a cryptococcal cell to another macrophage (Figure 1). Since this event was observed with both macrophage-like cells and primary murine macrophages, we decided to continue the studies in macrophage-like cells. This event was observed with all Cn strains used and when using 18B7 as the opsonin, but was not observed with HK Cn, beads or with macrophage-like cells treated with the actin-depolymerizing agent, cytochalasin D. In addition, the data shows the uninfected cells approaching the infected cell, fusing with it and acquiring the Cn cell from the infected cell (Figure 1), [see Additional file 1]. This transfer was often followed by the extrusion of the transferred cryptococcal cell.

**Figure 2:**
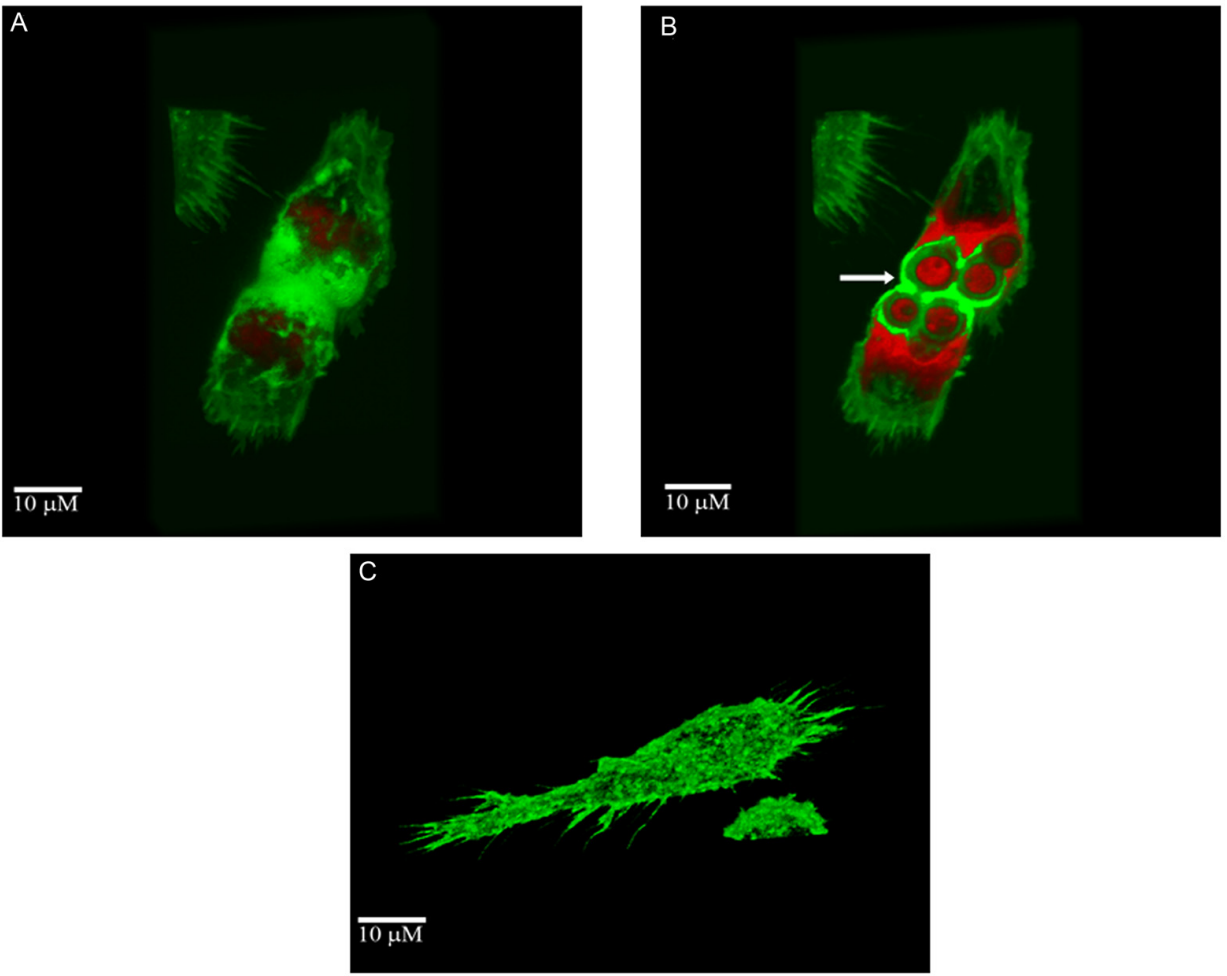
**Macrophage fusion and cell-to-cell Cn transfer involves actin**. 3D reconstructed confocal images show infected macrophages fusing (A). Cutting along the Z-axis of panel (A) demonstrates that the intracellular Cn are being shared by both macrophages and are surrounded by a high concentration of phalloidin-labeled actin from both macrophages, as indicated by the arrow (B). Panel (C) shows an uninfected macrophage. Images were collected at 63×.

The phenomenon of cell to cell transfer of Cn appeared to be dependent on the actin cytoskeleton since the uninfected macrophages generated cytoplasmic projections toward the infected cell that were not evident when macrophages were incubated with cytochalasin D. Using confocal microscopy to generate 3D images, we have observed that macrophages that contained Cn could fuse with one another, and when this occurred the Cn cell was surrounded by actin rich regions from both fusing macrophages (Figure 2).

**Figure 3:**
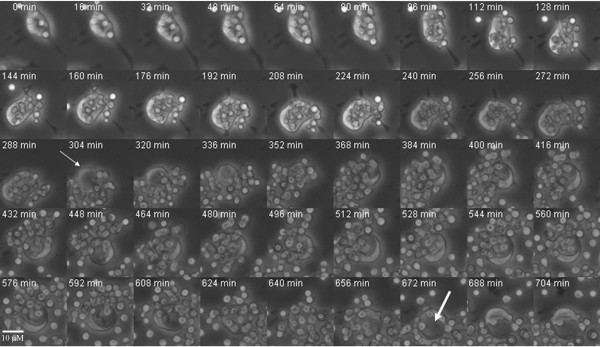
**Massive vacuole formation follows Cn extrusion by macrophages**. Macrophage extrusion of Cn cells was followed by the formation of a massive vacuole ranging up to approximately 15 μM in size. This formation was not observed in bead infected or uninfected macrophages. The thin arrow points to the nascent vacuole which progressively grows over time to a large vacuole, as indicated by the thick arrow. Images were collected at 10×.

In addition to cell-to-cell transfer of Cn cells, we witnessed another striking consequence to phagosome extrusion that involved the formation of a massive vacuole that ranged in sizes up to 10–15 μM within the macrophage-like cells that extruded the Cn cells (Figure 3). This event was not observed in uninfected macrophages or in macrophages where complement mediated phagocytosis of Cn cells or phagocytosis of beads (complement or Fc-mediated) had occurred. Massive vacuole formation generally occurred after complete extrusion of a high number of Cn cells '[see Additional file 2]'.

## Discussion

Cell-to-cell spread of intracellular pathogens is known to occur with viruses and bacteria such as HIV and *Shigella *spp., respectively [10-13]. Among the fungi, cell-to-cell spread could potentially occur when ingested cells form hyphae that protrude into neighboring cells, in a phenomenon that is associated with damage to both the donor and the host cell, although it is uncertain whether this has been described experimentally. Furthermore, we are not aware of any previous reports of cell-to-cell spread of yeast cells. Cell-to-cell spread is an important cellular pathogenic strategy because it allows cellular infection directly from the intracellular space and thus avoids exposure of the microbe to antimicrobial compounds in the extracellular space such as specific antibody and complement. Cell-to-cell spread can also protect microbes against certain antimicrobial drugs that have limited penetration into cells.

Cell-to-cell spread of *C. neoformans *was a relatively rare event. The phenomenon was observed with murine macrophages and macrophage-like cells under *in vitro *conditions, thus caution is warranted when extrapolating the relevance and importance of this phenomenon to *in vivo *situations. Nevertheless, the fact that it was observed provides the important precedent that cell-to-cell spread of yeast cells can occur. In this regard we note that in viral and bacterial systems cell-to-cell spread has also been documented only *in vitro*, yet is assumed to be possible *in vivo*.

*C. neoformans *cell-to-cell spread required contact between macrophage cells. The likely involvement of actin in this process was inferred by the observation that cell-to-cell transfer required the accepting cell to make contact with the donor cell through cytoplasmic projections that are abolished by the actin inhibitor cytochalasin D, in addition to confocal images shown in the results demonstrating actin rich regions from both fusing macrophages surrounding the cryptococcal cell. Although the mechanism for cell-to-cell transfer is unknown we note that cell membranes of infected cells appeared to be modified as evidenced by the extrusion of cryptococcal vacuoles with survival of the host cell and occasional cell-to-cell fusion events [5,6]. Hence, cell-to-cell transfer of *C. neoformans *may require microbial modification of the donor cell membrane followed by contact with an uninfected cell to create cell-to-cell interactions propitious for yeast transfer. This novel observation of Cn transfer from cell-to-cell raises the possibility that a similar mechanism could promote the dissemination of Cn during infection. For example, it is conceivable that cell-to-cell spread contributes to Cn dissemination in the CNS after crossing the blood-brain barrier. In addition to cell-to-cell spread, we report the formation of massive vacuole structures in macrophage-like cells that had previously harbored cryptococcal cells which had been released by the phenomenon of phagosome extrusion. Massive vacuole formation suggests that Cn intracellular habitation and replication in macrophage-like cells is associated with cellular damage, despite the fact that macrophages still remain alive as evidenced by their movement '[see Additional file 2]'. Perhaps longer imaging periods may have been needed to witness any cell death resulting from this event. The mechanism of massive vacuole formation is not known but we surmise that it may arise from the fusion of empty phagosomal remnants and perhaps polysaccharide containing vacuoles. In this regard, Cn phagosomal extrusion is associated with the retention of polysaccharide-containing vesicles that may then fuse with themselves or promote the fusion of other cellular membranes. Irrespective of the mechanism involved in massive vacuole formation, the presence of large vacuoles in previously infected cells provides evidence for the cytotoxicity of Cn intracellular residence. The occurrence of this event during infection could contribute to local tissue pathogenesis and promote Cn survival and persistence of infection.

## Conclusion

We describe two new phenomena, cell-to-cell spread of yeast cells and giant vacuole formation following extrusion by macrophages that harbor Cn. These observations highlight the dynamic nature of Cn interactions with macrophages and suggest the need for additional studies to establish the relevance of these events to the pathogenesis of *in vivo *cryptococcal infections.

## Methods

### Yeast strains and culture conditions

*C. neoformans var. gattii *(Serotype B) strain NIH 198 was provided by Thomas Mitchell (Durham, NC). *C. neoformans *var. *neoformans *strain 24067 was obtained from the American Type Culture Collection (Rockville, MD). *C. neoformans *var. *grubii *strain H99 was obtained from John Perfect (Durham, NC). All strains were cultured in Sabouraud dextrose broth (Difco) at 30°C with agitation (150–180 rpm). Additionally, when necessary Cn NIH 198 was killed by heating to 56°C (HK Cn) for 1 h and yeast death was confirmed by demonstrating no colony growth in Sabouraud agar.

### Murine macrophages and phagocytosis

The murine macrophage-like J774.16 cells, originally derived from a reticulum sarcoma [14,15], were used for all experiments. J774.16 cells were grown at 37°C with 10% CO_2 _in feeding media consisting of Dulbecco's minimal essential medium (DMEM) (Life Technologies), 10% NCTC-109 medium (Gibco), 10% heat-inactivated (56°C for 30 min) FCS (Gemini Bio-products, Woodland, CA, USA), and 1% non-essential amino acids (Mediatech Cellgro, Washington, DC, USA). For confocal microscopy, phenol red free DMEM (Gibco) supplemented with 2 mM L-glutamine was used. Cells were then plated on poly-lysine coverslip-bottom MatTek plates (MatTek Cultureware, Ashland MA) at a density of 5 × 10^4 ^per plate in feeding media, stimulated with 50 U/ml recombinant murine IFN-γ (Genzyme, Cambridge, MA) and incubated overnight at 37°C, 10% CO_2_. Phagocytosis assays were done as previously described [5,7]. Briefly, macrophage monolayers were incubated with a solution of Cn at a ratio of 10:1 effector to host (for vacuole formation studies) and 5:1 or 10:1 (for macrophage-macrophage transfer of Cn) along with 50 U/ml IFN-γ, 0.3 μg/ml LPS (Sigma, St. Louis, MO) and with 10–50 μg/ml of purified anti-capsular mAb 18B7 or 20% guinea pig serum (using H99 strain) for an additional 1 hr and subsequent microscopic time-lapse imaging was carried out.

For primary murine macrophage collection, 129SV (Jackson Laboratories, Bar Harbor, ME) 6–8 week old female mice were sacrificed by asphyxiation with CO_2_. Macrophages were collected by lavaging the peritoneal cavity with sterile PBS (10 ml washes per mice). Cells were pooled and spun down at 1200 rpm, counted, resuspended in feeding media aforementioned and plated in MatTek plates. Macrophages were allowed to adhere for at least 1 hr prior to incubation with Cn (using 18B7 as opsonin, as specified above) and subsequent microscopic imaging.

### Confocal microscopy

Prior to phagocytosis Cn was labeled with CellTracker™ Red CMTPX (Molecular Probes, Carlsbad, CA) at 37°C for 1 h, or 5-(and-6)-chloromethyl SNARF^®^-1, acetate (Molecular Probes) overnight shaking at 130–150 rpm at 30°C. Cn cells were thoroughly washed with PBS to eliminate residual amounts of probe and to prevent binding to intracellular macrophage proteins. Phagocytosis was carried out as indicated above and after 1 hr, macrophages and Cn were fixed with 4% paraformaldehyde, for 10 min followed by 5 min permeabilization with 1% triton-X 100 and finally labeling of actin on macrophages with Phalloidin-Alexa 488 (Molecular Probes). Samples were then suspended in mounting medium (50% glycerol and 50 mM *N*-propyl gallate in PBS) and visualized using a Leica AOBS laser scanning confocal microscope. Z-series images were collected using a 63×/1.4 Oil objective and 3D reconstructions were made using the Voxx software (Indiana University). Minor processing adjustments were made using Adobe Photoshop CS2.

### Time-lapse microscopy

For live cell imaging, phagocytosis was carried out as described above. Briefly, 5 × 10^4 ^macrophages were plated on polylysine coated coverslip bottom MatTek plates and allowed to adhere overnight. The media was then removed and replaced with fresh media containing Cn cells (Cn to macrophage ratio of 10:1 or 5:1), or anti-mouse IgG 3.2 μm beads (Spherotech) (effector to host ratio of 10:1) along with monoclonal antibody (mAb) against the cryptococcal capsule (mAb 18B7, 50 μg/ml for vacuole formation studies and, and 10–50 μg/ml for cell-cell spreading of Cn studies). The frequency of cell-to-cell transfer of Cn was determined by pooling all the data and taking a percentage of the number of events out of a total number of infected macrophages (500 total). In all phagocytosis experiments, macrophages were activated with 0.3 μg/ml lipopolysaccharide (LPS) (Sigma), and 50 units/ml of murine IFN-γ. Macrophages and Cn were incubated together for 60 min to allow for completion of phagocytosis, washed once with fresh media, replenished with 2 ml feeding media and followed by time-lapse imaging every 4 minutes. To assess whether the cytoskeleton was involved in cell to cell transfer of Cn, F-actin depolymerizing agent Cytochalasin D (Sigma) was added in feeding media 1/2 hr or 1 hr after phagocytosis was initiated at the inhibitory concentrations of 2 μM [16,17]. Images were collected at 10× or 20× using the Axiovert 200 M inverted microscope and photographed with an AxiocamMR camera controlled by the Axio Vision 4.4 software (Carl Zeiss Micro Imaging, NY). This microscope was housed in a Plexiglas box and the temperature was stabilized at 37°C with a forced air heater system. The plate lid was kept in place to prevent evaporation, and 5% CO_2 _was delivered to a chamber locally at the culture dish. Images were compiled into movies which were then used to analyze the Cn/macrophage interactions. Movie animations were created using ImageJ software [18].

## Note added in proof

This publication of this article was coordinated with the publication of the article 'Direct Cell-to-Cell Spread of a Pathogenic Yeast' by Ma et al in *BMC Immunology *[19].

## Authors' contributions

MA carried out all studies and drafted this manuscript under the tutelage, and mentorship of AC. AC contributed to drafting the manuscript. All authors read and approved the final manuscript.

## Supplementary Material

Additional file 1 **Cell to cell transfer of *C. neoformans *leads to infection of adjacent uninfected cell. **In this movie the cell to cell transfer of Cn is depicted. The uninfected macrophage located on the top left of the movie projects toward and acquires multiple Cn cells from the infected macrophage, resulting in its own infection, as indicated by the arrow. This event occurred in 2% of infected macrophages (data not shown). Significant frames were selected for the movies and were compiled at 7 frames per second. Images were collected at 20×. Click here for file

Additional file 2 **Extrusion of Cn cells by macrophages is followed by massive vacuole formation. **Extrusion of Cn by multiple macrophages, as shown in this movie, is followed by the formation of massive vacuoles on each macrophage that extruded. Movie compiled at 7 frames per second. Images were collected at 10×. Click here for file
